# Perceived readiness for hospital discharge: Patients with spinal cord injury versus physiotherapists

**DOI:** 10.4102/sajp.v74i1.437

**Published:** 2018-07-03

**Authors:** Marliza Du Plessis, Cassandra R. McGaffin, Thamsanqa Molepo, Roleen Oelofse, Susan Van Zyl, Mokgadi K. Mashola

**Affiliations:** 1 Department of Physiotherapy, University of Pretoria, South Africa

## Abstract

**Background:**

Successful discharge from rehabilitation for patients with spinal cord injury (PWSCI) relies on a smooth transition home. Assessing readiness for hospital discharge (RHD) is important in reducing secondary health conditions and improving satisfaction and function. Perception of PWSCI on RHD may be different from their physiotherapists, leading to difficulties.

**Objective:**

To compare the perceptions of PWSCI and physiotherapists with regard to RHD.

**Method:**

A comparative cross-sectional study included 50 PWSCI and their physiotherapists in Tshwane. They completed the Readiness for Hospital Discharge Scale (RHDS) and their responses to the subscales were compared. Data were analysed using descriptive and inferential statistics. Relationships between variables of interest and the general perception of RHD were determined using Pearson’s chi-square test. An independent samples *t*-test was used to analyse the difference in RHDS scores (including subscale scores) between PWSCI and physiotherapists. Results were significant if *p* < 0.05.

**Results:**

The total score of the RHDS was not significantly different (*t* = 1.31, df = 98, *p* = 0.19). Patients had higher perceptions in coping ability and expected support subscales (*t* = 3.15, df = 85.97, *p* = 0.002 and *t* = 4.23, df = 98, *p* = 0.0001, respectively). Physiotherapists had higher perceptions in the knowledge subscale regarding what to do and not do at home (*t* = -2.05, df = 82.08, *p* = 0.044) and follow-up sessions (*t* = 2.625, df = 85.28, *p* = 0.010).

**Conclusion:**

There was no difference in perception of readiness to go home, although physiotherapists gave lower scores for emotional readiness and ability to handle demands at home and higher scores for knowledge.

**Clinical implications:**

The use of the RHDS in the spinal cord rehabilitation units will better align the goals of rehabilitation and discharge planning to improve overall satisfaction with care and discharge outcomes. All members of a multidisciplinary team can achieve consensus and comparisons can be made on their patient’s perceived RHD.

## Introduction

Spinal cord injuries (SCIs) are debilitating and negatively affect the independence, lifestyle and quality of life of the patient (Middleton, Tran & Craig 2007). Spinal cord injuries are associated with impaired or loss of sensory or motor control. As a result, patients with spinal cord injury (PWSCI) have limited functional outcomes after rehabilitation, and readiness for discharge from rehabilitation is difficult to assess (Harvey 2016). Multidisciplinary teams (MDT), comprising of various health professionals, including medical officers, nurses and physiotherapists (Chhabra, Sharma & Arora 2018), have to set patient-specific rehabilitation goals in conjunction with the PWSCI, thus following a patient-centred approach. The main concerns and expectations of PWSCI have to be considered when attempting to improve their quality of life. Understanding how the perceptions of PWSCI differ from those of the MDT is important for rehabilitation, especially in light of major lifestyle changes (Simpson et al. 2012).

High-quality discharge education is linked to positive perceptions of readiness for hospital discharge (RHD); however, patients usually feel that they do not receive the necessary information for discharge while receiving too much unnecessary information (Maloney & Weiss 2008). A low perceived RHD is associated with not coping at home and an adverse post-discharge status, together with an increased rate of hospital readmissions (Maloney & Weiss 2008).

Patients with SCI should be rehabilitated using a patient-centred approach to assist them to reach goals pertaining to function, emotion and activities of daily living. Rehabilitation starts after PWSCI have been medically stabilised (Harvey 2016) and involves full participation from the patient and the MDT. Goals of rehabilitation are aimed at achieving expected functional outcomes, which consequently improve PWSCI’s independence and quality of life. Achieving these goals will influence their RHD (Mortenson, Noreau & Miller, 2010). Patients are deemed ready for hospital discharge after they have been educated about their health condition, how to manage and prevent secondary health conditions (SHCs) and how they are able to participate in the achievable activities of daily living as per their neurological levels of injury (Hassan, Visagie & Mji 2012). Readiness for hospital discharge may be delayed if there is disparity in the rehabilitation goals set by the MDT and patients’ desires and priorities (Draaistra et al. 2012). Disparities may arise if PWSCI are not adequately involved in the goal-setting process. The prioritised goals for rehabilitation should reflect both the PWSCI and their physiotherapists’ perceptions in deeming the patient ready for discharge (Mothabeng 2011).

Discharge from hospital and integration back into a residential environment can be limited by the development of SHCs, environmental barriers and personal factors (Mortenson et al. 2010). Secondary health conditions develop as a direct or indirect result of a primary disability such as SCI (Jensen et al. 2012). Secondary health conditions such as urinary tract infections, lung complications, gastrointestinal problems and pressure ulcers may result in readmission to hospital (Hammond et al. 2013). Readiness for hospital discharge may be influenced by barriers, which include a lack of funding for caregivers and home modifications and for necessary equipment to be able to function at home (New et al. 2013).

Gainful employment helps PWSCI achieve economic self-sufficiency and may be a source of adjustment to disability and life satisfaction. As a result, employment is one of the most important psychosocial aspects for PWSCI. However, the estimated employment rate in people with disabilities in South Africa is estimated at 25.2% (Pefile, Mothabeng & Naidoo 2016). Personal psycho-emotional factors may also affect RHD and functioning after discharge. Mothabeng et al. (2007) emphasised that PWSCI need to address emotions related to the injury and the effects of these emotions on social and family relationships.

Rehabilitation is an integral part of primary health care service delivery with only 24 specialised rehabilitation units available to PWSCI in sub-Saharan Africa, of which 16 are in South Africa (Southern African Spinal Cord Association 2017). To make optimal use of these facilities, PWSCI should not stay longer than necessary but should stay long enough to avoid readmission. Readmission may be attributed to inadequate pre-discharge preparation of the patient and family members. Patients with SCI who perceive themselves not to be ready for discharge may be unable to integrate into their residential environment (Mothabeng 2011). The inability to cope with the expected demands of independent function at home (Weiss, Yakusheva & Bobay 2010) leads to a higher risk of developing SHCs and consequently being readmitted to hospital (Hammond et al. 2013).

There is limited evidence on the perceptions of PWSCI and their physiotherapists regarding RHD, their individualised needs prior to discharge, as well as mutual rehabilitation goal setting. Thus, this study aimed to determine the perceptions of PWSCI and their physiotherapists on readiness for discharge.

## Method

We used a non-experimental, quantitative, cross-sectional comparative and descriptive design. Our study was set in two private hospitals and three public hospitals that admit patients with SCI in the Tshwane Metropolitan area.

All PWSCI (irrespective of the cause, level, type and completeness of injury) in the Tshwane Metropolitan area, who were being prepared for discharge and were older than 18, were included in the study. All participants had to be within seven days of discharge. Patients needed to be able to speak or understand any of the 11 South African national languages to be included in the study. The authors were able to speak and understand English, Afrikaans, Sepedi, Setswana, Sesotho and Zulu. A translator was present for participants who could not speak any of these languages. We used a non-probability, convenience sampling method. Fifty patients and their treating physiotherapists were included in our study.

### Procedure

Demographic data such as age, gender as well as injury profile including type and level of SCI were collected using a socio-demographic and injury profile questionnaire. Data pertaining to RHD were collected using the Readiness for Hospital Discharge Scale (RHDS). The RHDS is a self-report scale measuring the perception of a patient’s readiness to be discharged from hospital to a step-down facility or to the patient’s home (Weiss & Piacentine 2006).

The RHDS consists of 21 items and identifies four main subscale factors relating to a patient’s needs in the home setting after discharge: (1) personal status, (2) knowledge of their condition post-discharge, (3) coping ability once at home and (4) expected support at home (Weiss & Piacentine 2006). Items are scored on a 10-point Likert scale. Each measure was divided into four categories (Table 1) representing very high (9–10), high (8–8.9), moderate (7–7.9) and low (< 7) perceptions of discharge readiness (Weiss et al. 2014). The questions pertaining to pain and stress are reversely scored in the scale.

**TABLE 1:** The demographic characteristics of patients with spinal cord injury who were within seven days of being discharged (*n* = 50).

Demographics	Characteristics	Number	Percentage
Gender	Male	30	60
	Female	20	40
Age in years	18–29	12	24
	30–39	8	16
	40–49	14	28
	50–59	9	18
	> 60	7	14
Discharge setting	Home	41	82
	Rehabilitation setting	8	16
	Other	1	2
Discharged residential area	Township	17	34
	Suburb	20	40
	Informal settlement	7	14
	Other	6	12
Who do you live with?	Own family	48	96
	Relatives	1	2
	Other	1	2
Is help needed at home?	No	17	34
	Yes	33	66
Is there help at home?	Not applicable	15	30
	No	1	2
	Yes	34	68
Type of spinal cord injury	Paraplegia	35	70
	Tetraplegia	15	30
Level of spinal cord injury	C1–C4	5	10
	C5–T1	15	30
	T2–T6	11	22
	T7–T12	9	18
	L1–L5	9	18
	S1–S5	1	2
Completeness of spinal cord injury	Complete	11	22
	Incomplete	30	60
	Don’t know	9	18

The RHDS questionnaire takes approximately 5 to 10 min to complete and the PWSCI were interviewed after their therapy sessions, whereas the physiotherapists completed the questionnaire independently on the same day. The RHDS is considered to be reliable (Cronbach’s alpha, *α* = 0.90) and valid (Cronbach’s alpha, *α* = 0.82) in the adult medical-surgical, postpartum and parents of hospitalised children population (Weiss & Piacentine 2006). The RHDS has been validated for South African PWSCI (Cronbach’s alpha, *α* = 0.88) and physiotherapists (Cronbach’s alpha *α* = 0.93) (De Lange et al. 2017).

We contacted study settings weekly to identify the PWSCI selected for discharge from the hospital. The potential participants (both the PWSCI and their treating physiotherapists) were contacted, and the aims and objectives of the study were explained to them. The PWSCI and their treating physiotherapists were included in the study once informed consent was obtained. The PWSCI were given help with completing the RHDS questionnaires, given the variety of languages of the participants, and the physiotherapists completed the questionnaires on their own. The patient and their physiotherapist’s questionnaires were coded with the same numerals in order to link the responses. Data were collected from 01 February to 30 May 2017.

### Statistical analysis

Data were analysed with descriptive and inferential statistics, using SPSS v24. Socio-demographic information and the RHDS scores were analysed using frequencies, percentages, means and standard deviations. Relationships between variables of interest and the general perception of RHD were analysed using Pearson’s chi-square test. An independent samples *t*-test was used to analyse the difference in RHDS scores (including subscale scores) between PWSCI and physiotherapists. Results were significant if *p* < 0.05.

The interviewers collecting the information underwent training sessions to ensure that they asked the questions in the same way to ensure reliability of the questionnaire and underwent an internal team briefing on interviewing the patients to ensure internal validity.

### Ethical considerations

Institutional ethical clearance was obtained from the University of Pretoria (no. 474/2016). Written informed consent was obtained from all participants prior to participating in the study.

## Results

### Demographic data

In total, 50 patients and their treating physiotherapists participated in this study. The demographic information of the PWSCI in the study sample is shown in Table 1. There were more male PWSCI (60%, *n* = 30) than female PWSCI (40%, *n* = 20), mostly between 18 and 49 years of age (68%, *n* = 34). Most of the PWSCI were discharged home (82%, *n* = 41), with 98% (*n* = 48) living with family or relatives, whereas 16% (*n* = 8) were discharged to a rehabilitation setting and one patient to a care centre. The most common residential areas that the PWSCI were discharged to were suburbs (40%, *n* = 20) and townships (34%, *n* = 17). Thirty-three (66%) PWSCI reported that they would need help at home. The majority of the PWSCI had paraplegia (70%, *n* = 35), while only 10% had a level of SCI between C1 and C4 (*n* = 5). Sixty per cent of the PWSCI had incomplete injuries (*n* = 30).

### General readiness for hospital discharge

**FIGURE 1:**
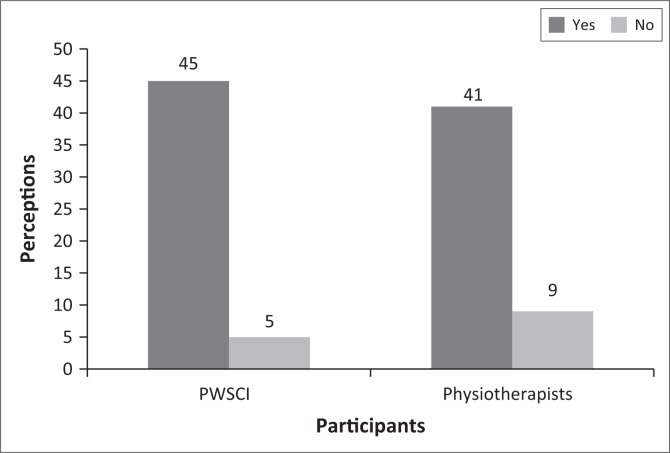
Perceptions of general readiness for hospital discharge for patients with spinal cord injury (PWSCI) and physiotherapists (*n* = 50).

The first item in the RHDS questionnaire was a general gauge of RHD with the question: ‘As you think about your planned discharge from the hospital, do you believe that you are ready to go home as planned?’ to which a yes or no answer was given. Forty-five (90%) PWSCI responded yes to general RHD with *n* = 5 (10%) responding no, whereas 41 (82%) physiotherapists responded yes to general RHD and the other 9 (18%) responded no (Figure 1). A Pearson’s chi-square test showed no significant difference in the response (*χ*^2^ = 1.329, df = 1, *p* = 0.249).

**FIGURE 2:**
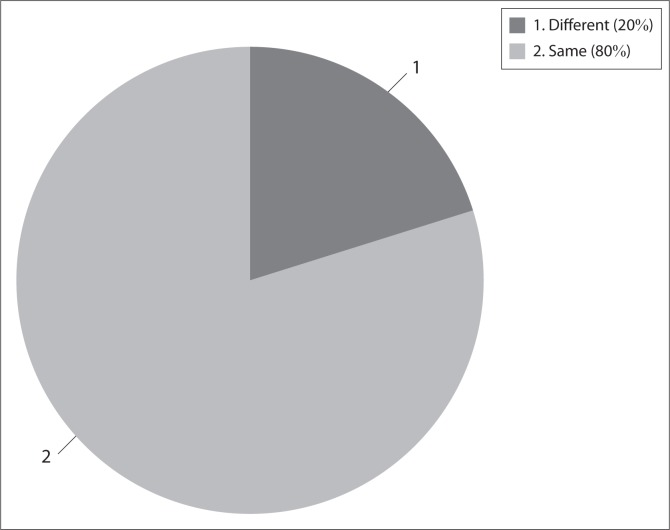
Agreement between patients with spinal cord injury and their physiotherapists regarding their readiness for hospital discharge (percentage).

Perceptions of general RHD between groups (physiotherapists and their PWSCI) were similar in 80% of the cases (Figure 2). Only two PWSCI (4%) agreed with their physiotherapists that they were not RHD. Three PWSCI perceived that they were not ready for discharge but their physiotherapists perceived that they were ready to be discharged.

Table 2 depicts results of the RHDS. Patients with SCI had a total RHDS mean score of 150.78 (SD = 27.06) and physiotherapists’ total mean score was 143.38 (SD = 29.29). The difference was not statistically significant (*t* = 1.31, df = 98, *p* = 0.19). The coping ability and expected support subscales showed significantly higher perceptions of PWSCI than the physiotherapists (*t* = 3.15, df = 85.97, *p* = 0.002 and *t* = 4.23, df = 98, *p* = 0.000, respectively). Patients had higher perceptions concerning their ability to handle demands at home (*t* = 2.41, df = 98, *p* = 0.018), to perform self-care (*t* = 2.11, df = 98, *p* = 0.038) and to perform medical treatments (*t* = 3.51, df = 89.98, *p* = 0.001) (Table 3).

**TABLE 2:** Readiness for Hospital Discharge Scale (RHDS) scores for patients with spinal cord injury and their attending physiotherapists.

Items in RHDS	Variable	Patients with spinal cord injury		Physiotherapists	

		Mean	Standard deviation	Mean	Standard deviation
Personal status	Physical readiness*	7.86	2.04	6.66	3.00
	Pain or discomfort	2.46	2.70	2.56	1.90
	Strength	6.96	1.98	6.64	2.20
	Energy	7.14	2.11	6.70	2.30
	Emotional readiness*	8.48	2.37	7.14	2.70
	Physical self-care	7.14	2.28	6.52	3.00
	Stress*	2.46	2.64	4.04	2.48
**Subscale score**	**-**	**42.50**	**7.73**	**40.26**	**11.19**
Knowledge	Self-care	7.52	2.72	8.08	2.00
	Medical needs	7.88	2.44	7.90	2.00
	Problems to watch out for	7.68	2.36	8.14	1.70
	Who and when to call when problems arise	7.28	2.86	8.22	1.90
	Restrictions on what to do (not do)*	7.24	2.70	8.16	1.70
	Follow up*	6.24	3.27	7.70	2.20
	Services and general information	6.60	3.15	6.54	2.10
**Subscale score**	**-**	**50.44**	**14.35**	**54.74**	**11.11**
Coping ability	Handle demands at home*	7.42	2.34	6.28	2.40
	Performing personal care*	7.70	2.05	6.70	2.70
	Medical treatment*	8.22	2.12	6.44	2.90
Subscale score*	**-**	**23.34**	**4.92**	**19.42**	**7.29**
Expected	Emotional support*	9.14	1.31	7.64	7.30
support	Help with personal care*	8.80	1.80	7.64	1.80
	Household activities*	8.26	2.23	6.92	2.30
	Medical needs*	8.30	2.01	7.16	2.10
Subscale score*	**-**	**34.50**	**6.15**	**28.96**	**6.93**

**Total RHDS score**		**150.78**	**27.06**	**143.38**	**29.29**

* , statistically significant differences (see Table 3).

**TABLE 3:** Significant differences of Readiness for Hospital Discharge Scale subscale scores between patients with spinal cord injury and physiotherapists.

Variables	*T*	df	Mean difference	95% confidence interval		*p*

				Lower	Upper
Coping ability total score	3.15*	85.97	3.92	1.45	6.39	0.002
Handle demands at home	2.41	98.00	1.14	0.20	2.08	0.018
Performing personal care	2.11	98.00	1.00	0.06	1.94	0.038
Medical treatment	3.51*	89.98	1.78	0.77	2.79	0.001
Expected support total score	4.23	98.00	5.54	2.94	8.14	0.000
Emotional support	4.72*	88.84	1.50	0.87	2.13	0.000
Help with personal care	4.55	98.00	1.88	1.06	2.70	0.000
Household activities	2.25	98.00	1.10	0.24	1.96	0.013
Medical needs	2.70	98.00	1.06	0.28	1.84	0.008
Physical readiness	2.35	98.00	1.20	0.19	2.21	0.021
Emotional readiness	2.65	98.00	1.34	0.34	2.34	0.009
Stress	−3.08	98.00	−1.58	−2.60	−0.56	0.003
Restrictions on what to do (not do)	−2.05*	82.08	−0.92	−1.82	−0.03	0.044
Follow up	2.63*	85.28	−1.46	−2.57	−0.35	0.010

Independent samples *t*-test is significant if *p* < 0.05.

* , Levene’s test is *p* < 0.05, equal variance not assumed.

df, degrees of freedom.

For the expected support subscale, PWSCI had high perceived readiness for help with personal care (*t* = 4.55, df = 98, *p* = 0.000) and felt that they would receive enough emotional support at home (*t* = 4.72, df = 88.84, *p* = 0.000) compared to physiotherapists. Patients also had a high perception of help with household activities (*t* = 2.25, df = 98, *p* = 0.013) and medical needs (*t* = 2.70, df = 98, *p* = 0.008).

Although the personal status subscale score was not significantly different between PWSCI and physiotherapists, three of the seven items were found to be significantly different between the groups. Patients perceived themselves to be both physically and emotionally ready for hospital discharge but their treating physiotherapists did not (*t* = 2.35, df = 98, *p* = 0.021 and *t* = 2.65, df = 98, *p* = 0.009, respectively). Physiotherapists perceived higher stress levels than the patients themselves (*t* = -3.08, df = 98, *p* = 0.003).

In the knowledge subscale, physiotherapists perceived that their patients had adequate knowledge on what they are allowed (and not allowed) to do at home (*t* = -2.05, df = 82.08, *p* = 0.044), as well as knowing what happens in their next follow-up session (*t* = 2.625, df = 85.28, *p* = 0.010).

## Discussion

Although both PWSCI and their treating physiotherapists had similar perceptions of the general RHD, the RHDS showed significant differences in specific aspects relating to the patient’s readiness in being discharged from the hospital. General RHD can be influenced by both the PWSCI and physiotherapist knowing that the patient is only at the rehabilitation facility for a specified period of time. Once the specified time has arrived, both parties may feel that the patient is generally ready for hospital discharge (Weiss & Piacentine 2006). Despite the general RHD, the responses were not overwhelming, with the PWSCI and their physiotherapists reporting moderate overall readiness for discharge on the RHDS. This finding may be attributed to the fact that patients in South Africa are being discharged from rehabilitation before being ready for community reintegration, as suggested by Mothabeng (2011).

Adjusting to a new reality may impact the transition from the rehabilitation centre to home, which is regarded as the greatest challenge for PWSCI (Mothabeng 2011). Uncertainty of what to expect at home and whether or not adequate support is available is one of the main barriers to community reintegration in the Tshwane Metropolitan area (Mothabeng 2011). Most PWSCI in our study perceived that they would need help at home after discharge. Despite this, most felt that they would be able to handle the demands of life, to perform self-care and to perform medical treatments, whereas physiotherapists felt that their patients were not ready to handle such demands. Patients with SCI may overestimate their coping ability and support they expect to receive at home because they are in a safe rehabilitation environment, surrounded by patients with similar conditions and have full-time access to health care. The PWSCI may not be aware of all the physical or emotional requirements once discharged, as many patients are discharged before reaching functional independence (Hastings, Ntsiea & Olorunju 2015). The yearning to be home may play a role in patient’s perceptions of being emotionally ready to be discharged. The perceptions of stress felt by PWSCI differed between the two groups in our study. It is possible that PWSCI do not fully disclose their concerns and fears about discharge. Physiotherapists, having more experience in rehabilitation, may be more aware of how emotional states can influence their patients’ quality of life, especially the first three months after discharge (Mortenson et al. 2010), and therefore overestimate their patient’s stress levels.

In the knowledge subscale, physiotherapists and PWSCI differed in their perception of knowledge for follow up treatments and what the patient was allowed to do, and not do once discharged. This finding is supported by Weiss et al. (2010), who found that health workers tend to overestimate medical and surgical patients’ knowledge of their post-discharge plan. This finding suggests that physiotherapists may consider that patients successfully interpret all information taught, which may not be the case. Patients may feel overwhelmed at home once they are without the health care professionals they were accustomed to while undergoing rehabilitation. Many patients may experience uncertainty about their medical condition, what to do and how to take their medications (Coffey & McCarthy 2012). It is important for PWSCI to have the adequate information necessary to cope at home and to prevent SHCs once home.

The expected support subscale showed that patients had a high perception of readiness for help with household activities and help with medical care and emotional support. Physiotherapists, in contrast, had a low perception of readiness for help with household activities and a moderate readiness perception with medical care and emotional support. Patients with SCI may have higher expectations for recovery and thus not be aware of the challenges they face upon discharge, and may underestimate the amount of support they need (Wiles et al. 2002).

Knowledge of the differences in perceptions of PWSCI and their physiotherapists can be used to align rehabilitation outcomes with the needs of the PWSCI. This knowledge can also be used to develop improved discharge teaching strategies (Weiss et al. 2010). Education programmes during rehabilitation may be restructured to better suit each patient’s profile (such as emotional state, duration after injury and relevance of education at the time of injury). Patients may experience ‘information overload’ and be unable to sift through all the information when the time to use the knowledge presents itself. Rehabilitation efforts should also focus on preparing PWSCI for the demands of life at home and enable them to perform their own medical treatments and maintain self-care. It is possible that PWSCI overestimate their readiness for discharge and therefore score themselves higher than their physiotherapists because they are eager to be discharged from the hospital setting. Physiotherapists may need to delve deeper into the social support structure to ensure that they do not underestimate the expected help when the PWSCI returns home. Differences in perception of RHD between PWSCI and their physiotherapists may imply that PWSCI need even more home visits prior to discharge to help adjust to their home environment. Family meetings may also be scheduled more often to establish available support once home. Physiotherapists may need to revise information learnt during rehabilitation with PWSCI prior to discharge.

The limitations of the study include a small sample as well as it being a sample of convenience. The study only focused on PWSCI who were admitted to rehabilitation facilities in the Tshwane Metropolitan area. This study did not determine demographic information of physiotherapists, therefore could not establish whether their work experience and expertise could be linked to a difference in perceptions.

We recommend that the RHDS be included by the MDT in the rehabilitation of PWSCI, to ensure better alignment of the goals of rehabilitation and discharge planning to improve overall satisfaction with care and discharge outcomes (Knier et al. 2015; Weiss et al. 2010). We recommend that each member of the MDT use the RHDS to reach consensus on whether they deem the PWSCI ready for discharge and compare their findings to the patient’s own perception. It is also recommended that future studies identify a possible relationship between RHD and subsequent readmissions after discharge in PWSCI.

## Conclusion

Patients with SCI and their treating physiotherapists have similar perceptions of their general RHD. Their perceptions of readiness differ regarding the PWSCI’s coping ability and expected support once discharged. Emphasis should be placed on the above-mentioned aspects during rehabilitation, to better equip PWSCI with coping strategies and to put systems in place for optimum support once discharged. By so doing, difficulties with post-discharge coping may be alleviated and ultimately reduce the occurrence of hospital readmission.
